# A cooperative-binding split aptamer assay for rapid, specific and ultra-sensitive fluorescence detection of cocaine in saliva†
†Electronic supplementary information (ESI) available: Optimization of Mg^2+^ and ATMND concentrations for our CBSA-based ATMND-binding assay; ATMND-reported calibration curve for CBSA-5325 at various cocaine concentrations; ATMND binding affinity for the cocaine-assembled CBSA-5325; *K*_D_ of 38-GC and different 38-GC mutants for cocaine as characterized by ITC; stem length effects on cocaine-induced CBSA assembly; spectra of CBSA-5335-based fluorescence detection of cocaine in 1× binding buffer; characterization of cocaine binding affinity of CBSA-5335 and PSA using ITC; fluorescence detection of cocaine in saliva with our fluorophore/quencher modified CBSA-5335; calibration curve of our CBSA-5335-based fluorophore/quencher assay in 1× binding buffer and 10% saliva at cocaine concentrations ranging from 0 to 10 μM; bias and precision of the CBSA-5335-based fluorophore/quencher assay; comparison of amplification-free split-aptamer assays for cocaine detection; sequence ID and DNA sequences used in this work. See DOI: 10.1039/c6sc01833e



**DOI:** 10.1039/c6sc01833e

**Published:** 2016-07-29

**Authors:** Haixiang Yu, Juan Canoura, Bhargav Guntupalli, Xinhui Lou, Yi Xiao

**Affiliations:** a Department of Chemistry and Biochemistry , Florida International University , 11200 SW 8th Street , Miami , FL 33199 , USA . Email: yxiao2@fiu.edu; b Department of Chemistry , Capital Normal University , Xisanhuan North Rd. 105 , Beijing , 100048 , China

## Abstract

Sensors employing split aptamers that reassemble in the presence of a target can achieve excellent specificity, but the accompanying reduction of target affinity mitigates any overall gains in sensitivity. We for the first time have developed a split aptamer that achieves enhanced target-binding affinity through cooperative binding. We have generated a split cocaine-binding aptamer that incorporates two binding domains, such that target binding at one domain greatly increases the affinity of the second domain. We experimentally demonstrate that the resulting cooperative-binding split aptamer (CBSA) exhibits higher target binding affinity and is far more responsive in terms of target-induced aptamer assembly compared to the single-domain parent split aptamer (PSA) from which it was derived. We further confirm that the target-binding affinity of our CBSA can be affected by the cooperativity of its binding domains and the intrinsic affinity of its PSA. To the best of our knowledge, CBSA-5335 has the highest cocaine affinity of any split aptamer described to date. The CBSA-based assay also demonstrates excellent performance in target detection in complex samples. Using this CBSA, we achieved specific, ultra-sensitive, one-step fluorescence detection of cocaine within fifteen minutes at concentrations as low as 50 nM in 10% saliva without signal amplification. This limit of detection meets the standards recommended by the European Union's Driving under the Influence of Drugs, Alcohol and Medicines program. Our assay also demonstrates excellent reproducibility of results, confirming that this CBSA-platform represents a robust and sensitive means for cocaine detection in actual clinical samples.

## Abstract 2

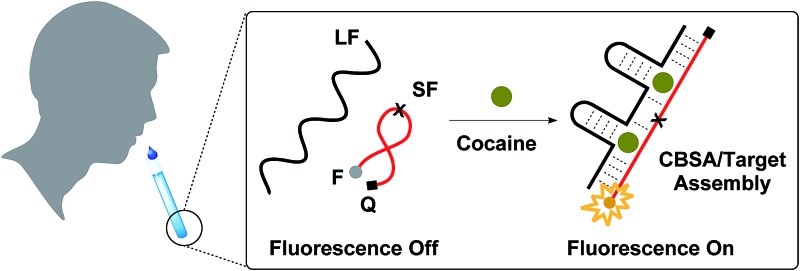
A fluorescence assay based on a split aptamer featuring a cooperative-target-binding mechanism performs one-step, rapid detection of as low as 50 nM in 10% saliva without signal amplification.

## Introduction

Aptamers are single-stranded oligonucleotides isolated *via in vitro* systematic evolution of ligands by exponential enrichment (SELEX),^1,2^ which can specifically bind to a wide range of targets including proteins, small molecules and metal ions.^3^ Aptamers offer several advantages as recognition elements for biosensor applications relative to antibodies. First, aptamers can be chemically synthesized with high reproducibility at relatively low cost.^4,5^ Second, the high chemical stability of DNA aptamers means that they can be used under harsher conditions and stored with a longer shelf life.^6^ Finally, it is possible to generate unstructured aptamers that form specific secondary structures such as three-way junctions^7,8^ or tertiary folds such as G-quadruplex structures^9,10^ upon target binding. Such target-induced conformational changes can be readily exploited for specific target detection in a variety of applications, including medical diagnostics, environmental monitoring and drug screening.^11–13^

However, target-induced conformational change is hard to control, especially for small-molecule-binding aptamers that have relatively high dissociation constants (∼μM *K*_D_).^14^ For example, the well-characterized cocaine-binding aptamer MNS-4.1 (*K*_D_ ∼ 5 μM) is structurally stable and forms a three-way junction even before binding cocaine.^15^ To achieve an effective target-induced conformational change, Stojanovic *et al.* truncated the sequence to destabilize the aptamer, so that it exists in an equilibrium state consisting of both folded and unfolded structures.^7^ This aptamer exhibited cocaine-induced folding, but still retained some folding activity in the absence of target, resulting in a high background that significantly limited sensor sensitivity.^7,16^ This background can be reduced by splitting the aptamer into two^17^ or three^18^ fragments, which further destabilizes the aptamer such that the fragments are unable to assemble without target. This results in minimal background signal,^17,18^ while the fragments retain their capacity for target recognition and can successfully reassemble into a complex secondary structure in the presence of cocaine. However, aptamer splitting results in notably reduced target affinity.^17^ Thus, this approach still compromises the sensitivity of split aptamer-based sensors.

To improve the sensor's sensitivity, Zhang *et al.* reported an assay based on an enzyme-linked split aptamer to perform colorimetric cocaine detection.^19^ One of the fragments was conjugated to a plastic surface; in the presence of cocaine, this fragment would form a complex with the second fragment, which was modified with biotin in order to bind streptavidin-linked horseradish peroxidase for signal amplification. However, washing caused the dissociation of a subset of the assembled tripartite complexes, resulting in a limit of detection (LOD) of just 2.8 μM. To address this, Heemstra *et al.* incorporated a proximity ligation strategy into this enzyme-amplified split-aptamer-based assay.^20^ Specifically, cocaine binding facilitated the assembly of the two azide- and cyclooctyne-modified split fragments, bringing these two chemical groups into close proximity such that covalent bonds could be formed. This prevented dissociation of the assembled target–aptamer complex during washing and improved the sensor's detection performance. Although the signal was amplified by the enzyme, the sensitivity remained limited to 0.1 μM^21^ due to the low binding affinity of the split aptamer (*K*_D_ = ∼200 μM).^17^ To overcome this limitation, we have developed a cooperative binding-based approach to generate split aptamers that retain high target affinity. Cooperative binding behavior is commonly observed in ligand-binding proteins that are highly responsive to ligand concentration, such as hemoglobin,^22^ ion channels^23^ and transcription factors.^24^ Those proteins generally have more than one ligand-binding site, where binding at one site increases the affinity of the other sites, resulting in a ‘switch-like’ binding curve.^25^ Breaker *et al.* initially found that some tandem riboswitches^26,27^ naturally employ such cooperative binding^26^ to control gene expression in response to subtle changes in ligand concentration. This cooperative behavior was further extended into artificial biosystems such as ribozymes,^28^ molecular beacons^29^ and DNA aptamers.^30^ Specifically, Plaxco *et al.* introduced the disorder into the parent aptamer to achieve cooperative binding, and focused on the demonstration of the “switch-like” response behavior of cooperative binding that occurred when ligand concentration approaches *K*_1/2_ (*K*_1/2_ represents the ligand concentration at which half of the binding domains are occupied). These engineered cooperative DNA aptamers could not be employed for a practical sensor platform because the introduction of cooperativity unavoidably reduced the target-binding affinity of the resulting cooperative aptamer (*K*_1/2_ = ∼3 mM).^30^

We have for the first time successfully incorporated two tandem target-binding domains into a split aptamer (termed cooperative binding split aptamer, CBSA) to achieve more sensitive detection of cocaine, even in complex biological samples. The initial cocaine-binding event stabilizes the structure of the split aptamer and facilitates subsequent target-binding at the second binding domain. Our CBSA exhibits higher target affinity and far more responsive target-induced aptamer assembly compared to the single-domain parent split aptamer (PSA) from which it was derived. Using a fluorophore/quencher pair, we have demonstrated that a CBSA-based fluorescence assay can achieve sensitive and reproducible cocaine detection in biofluid specimens, with a LOD of 50 nM in 10% saliva within 15 min. Given the simplicity of splitting and engineering a CBSA from an isolated aptamer and the excellent performance of the CBSA-based assay in biofluid samples, it should be straightforward to develop other CBSA-based assays from existing or future aptamers for rapid, sensitive and specific detection of various targets in clinical or field settings in a simple, low-cost assay format.

## Results and discussion

**Fig. 1:**
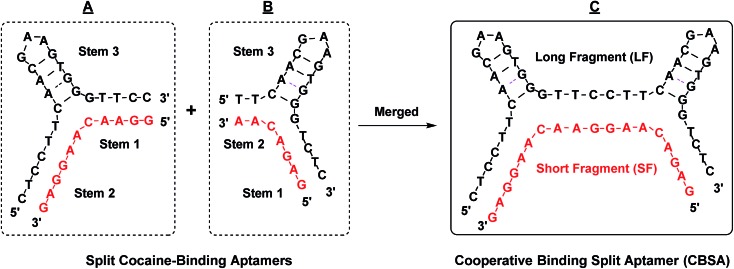
The design of our cocaine-binding CBSA. Two split aptamer pairs were derived from 38-GC. Stem 1 of one split aptamer (A) was merged with stem 2 of the other split aptamer (B) to form an engineered CBSA (C) comprising a short fragment (SF) and a long fragment (LF).

Cooperative target binding of split aptamers requires the incorporation of at least two binding domains into a single pair of fragments. We previously reported a cocaine-binding aptamer (38-GC)^31^ that exhibits 2.5-fold higher cocaine affinity than the MNS-4.1 aptamer from which it was derived. 38-GC contains a three-way junction with the target-binding domain located at its center, surrounded by three double-stranded stems (stems 1, 2 and 3) and two loops (GAA and AAA loops). We have determined that stem 3 is essential for cocaine binding, while both stem 1 and stem 2 contribute to the stability of the three-way junction structure that forms upon target binding.^31^ We derived two different pairs of split aptamer fragments from 38-GC (Fig. 1A and B), in which stem 3 remained intact but the 3′-end of stems 1 and 2 was truncated and the AAA loop of stem 2 was eliminated. We subsequently produced a construct from these two sets of split aptamers, in which stem 1 from one set was linked to stem 2 from the second set (Fig. 1C). The resulting CBSA consists of a short fragment (SF) and a long fragment (LF).

**Fig. 2:**
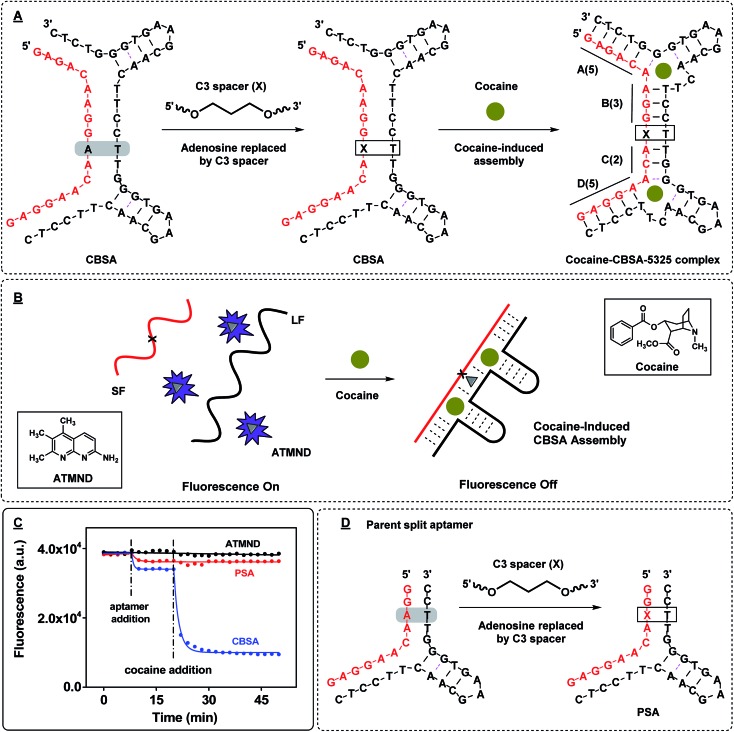
ATMND as a fluorescence reporter of target-induced CBSA assembly. (A) We modified the core CBSA sequence (left) by replacing the adenosine (at position 10 from 5′) between the two binding domains of the short fragment with a C3 spacer abasic site (marked as X) to yield the final CBSA construct (middle). Upon binding cocaine, the CBSA undergoes target-induced assembly, forming a dinucleotide bulge within each three-way junction (right). The cocaine–CBSA-5325 complex features four complementary double-stranded regions (A–D) and a duplexed AP site. (B) In the absence of cocaine, LF and SF remain separated and the unbound ATMND in solution generates a strong fluorescence signal. Cocaine induces CBSA assembly, forming the duplexed AP site that binds ATMND and thereby quenches its fluorescence. (C) Time-course of ATMND quenching by cocaine-induced split aptamer assembly. (D) Sequence of the parent split aptamer (PSA) with incorporated AP site.

We anticipated that the CBSA fragments would remain separated in the absence of target, but would form two tandem cocaine-binding domains when fully assembled with cocaine. To confirm effective target-induced CBSA assembly, we developed a binding assay based on the fluorescent molecule 2-amino-5,6,7-trimethyl-1,8-naphthyridine (ATMND). It has been reported that ATMND can strongly bind to a thymine situated opposite a C3 spacer abasic site (AP site) within a DNA duplex (*K*_D_ = 111 nM) *via* three-point hydrogen bonding.^32^ Although ATMND fluoresces brightly when free in solution, this fluorescence is greatly quenched when ATMND is bound to a DNA duplex in this fashion.^33^ We therefore replaced the adenosine (at position 10 from 5′) between the two binding domains of the short fragment with a C3 spacer to form an AP site with a thymine in the opposite position within the long fragment upon cocaine binding (Fig. 2A). In the absence of cocaine, the long and short fragments remain separated, with strong fluorescence produced by the free ATMND molecules (Fig. 2B, left). Upon addition of cocaine, the CBSA fragments undergo cooperative target-induced assembly and form a duplexed AP site that binds ATMND and quenches its fluorescence (Fig. 2B, right). The resulting CBSA-5325/cocaine complex contains four complementary base-paired segments (Fig. 2A, right; segments labeled (A)–(D)) and a dinucleotide bulge formed within each three-way junction.^34^

We subsequently confirmed the cocaine-induced assembly of CBSA experimentally. When we mixed 1 μM each of the long and short fragments with 200 nM ATMND in 1× binding buffer (10 mM Tris–HCl, 100 μM MgCl_2_, pH 7.4), we observed 10% background quenching (Fig. 2C, before cocaine addition). This quenching is most likely attributable to low levels of non-specific quench and non-target assembly of the CBSA. Upon addition of 250 μM cocaine, 72% of the ATMND fluorescence was quenched within 15 min, indicating rapid target-induced CBSA assembly (Fig. 2C, after cocaine addition). We also tested the performance of the AP-incorporating parent split aptamer (PSA) from which the CBSA was initially derived (Fig. 2D, PSA), which features only a single cocaine-binding domain, in our ATMND-binding assay. In the absence of cocaine, the PSA quenched 6% of ATMND fluorescence (Fig. 2C, before cocaine addition), and no measurable signal change was observed upon addition of 250 μM cocaine (Fig. 2C, after cocaine addition). This indicates that no cocaine-induced assembly is taking place, presumably because the shortening of stem 1 to four base-pairs with an abasic site in the middle results in poor thermodynamic stability of the cocaine–PSA complex at room temperature. We therefore conclude that the quenching observed most likely arises through non-specific interactions between PSA and ATMND.

**Fig. 3:**
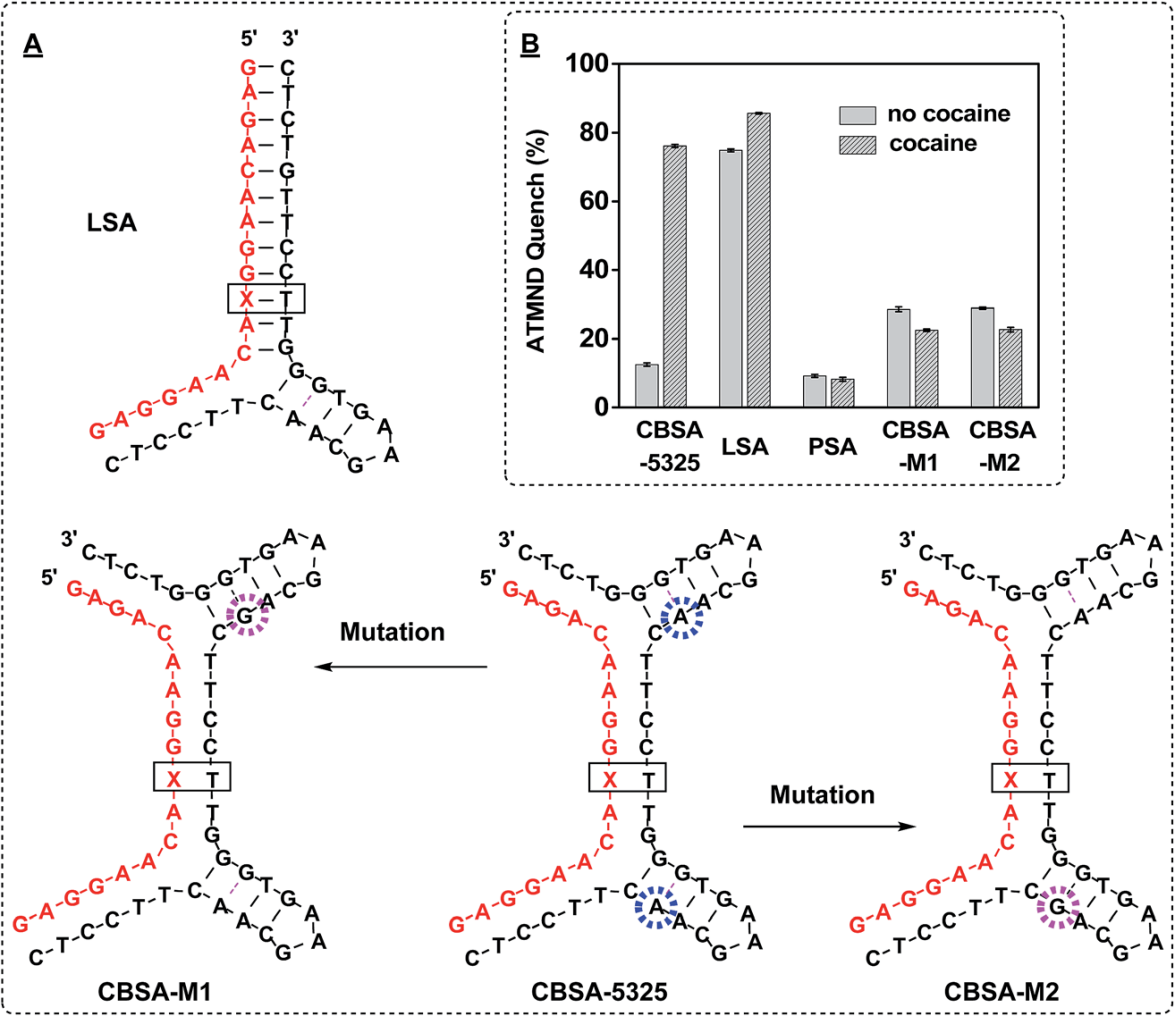
Analyzing the dual binding domains of CBSA for target-induced assembly. (A) We generated multiple derivatives of CBSA-5325, including split aptamers with a single binding pocket (LSA) and a pair of point-mutants (CBSA-M1 and CBSA-M2) with sequence alterations that disrupt either of the two binding domains (pink circle). (B) ATMND quenching in the presence or absence of 250 μM cocaine. Quenching was calculated by (*F*_A_ – *F*)/*F*_A_ × 100%, where *F*_A_ is the fluorescence of 200 nM ATMND in 1× binding buffer alone and *F* is the fluorescence of the ATMND–CBSA mixture with 250 μM cocaine or without cocaine, respectively.

Aptamer binding affinity^7,35^ and DNA hybridization efficiency^36^ are both strongly affected by magnesium concentration. We therefore used our ATMND-binding assay to optimize cocaine-induced CBSA assembly by varying the Mg^2+^ concentration from 0 to 1000 μM, and observed that maximum cocaine-induced ATMND quenching occurred in the presence of 100 μM Mg^2+^ (ESI, Fig. S1A†). In the absence of cocaine, we observed 9% quenching without Mg^2+^, which we attributed to non-specific CBSA assembly (ESI, Fig. S1A,† no cocaine). The presence of cocaine only generated an additional 7% quenching due to the absence of the Mg^2+^ counterion, resulting in strong repulsion between the CBSA fragments (ESI, Fig. S1A,† cocaine). At high concentration of Mg^2+^ (1000 μM), extensive CBSA assembly occurred in the absence of cocaine, producing 38% ATMND quenching, while also reducing the binding affinity of CBSA to cocaine, which only produced an additional 17% quenching upon addition of 250 μM cocaine (ESI, Fig. S1A†). We also varied the ATMND concentration from 50 to 1000 nM, and found that 200 nM ATMND produced low background and high target-induced signal change (ESI, Fig. S1B†). Under these optimized conditions (200 nM ATMND and 100 μM Mg^2+^), we examined the extent of target-induced CBSA assembly at different cocaine concentrations. We found a strong correlation between ATMND quenching from CBSA assembly and cocaine concentration in the range of 0 to 250 μM (ESI, Fig. S2†). We characterized the binding affinity of the assembled-CBSA for ATMND by titrating different concentrations (0–20 μM) of CBSA into 200 nM ATMND in the presence of 1 mM cocaine (ESI, Fig. S3†). The calculated *K*_D_ was 365 nM, which is consistent with the reported value for ATMND binding to AP sites.^32^ We subsequently demonstrated that the presence of two binding sites makes CBSA-5325 far more responsive to the presence of cocaine than split aptamers containing a single binding domain. We produced a long split aptamer (LSA) from CBSA, in which we replaced one of the binding domains with fully complementary sequences (Fig. 3A, LSA). The LSA fragments quenched 75% of ATMND fluorescence in the absence of cocaine. Addition of 250 μM cocaine only induced an additional 10% signal change, indicating that most LSA fragments were stably pre-assembled even without target (Fig. 3B). In contrast, CBSA-5325 generated a large signal change (72%) upon addition of 250 μM cocaine because of its dual target binding domains, with far weaker background signal (9%) without cocaine.

Specific target binding at both domains is required for target–CBSA assembly. We tested the binding affinity of 38-GC and several point-mutated derivatives using isothermal titration calorimetry (ITC) (ESI, Fig. S4†). We found that replacing an adenosine at position 22 with guanine completely impaired cocaine binding (ESI, Fig. S4,† 38-GC-22G). Based on this finding, we created two CBSA mutants (CBSA-M1 and CBSA-M2) in which either of the two binding domains was disrupted by this single-nucleotide mutation, leaving only a single binding domain capable of binding cocaine (Fig. 3A). CBSA-M1 and CBSA-M2 were mutated at the 3′- and 5′-binding domain of the long fragment, respectively. We tested these mutants using the same ATMND-binding assay, and found that neither CBSA-M1 nor CBSA-M2 was capable of cocaine-induced aptamer assembly, with no significant ATMND quenching observed upon addition of cocaine (Fig. 3B). These results confirmed that both target-binding domains of the CBSA were required for target-induced aptamer assembly, and thus provide strong support for a cooperative-binding-based assembly mechanism.

We hypothesized that the binding affinity of the CBSA might be enhanced by further stabilizing the target/aptamer complex with additional base-pairs, based on prior findings that longer complementary stems surrounding the three-way junction increased the aptamer's target-binding affinity.^37^ Thus, we increased the total number of base-pairs between the two binding domains of CBSA-5325 by adding an additional one, two or three base-pairs to generate CBSA-5335, -5435 and -5445 (ESI, Fig. S5A†) and examined the extent of target-induced CBSA assembly at different cocaine concentrations (0–50 μM) using our ATMND-binding assay. Our results demonstrated that the number of additional base-pairs greatly affects both binding affinity and target-induced assembly. In the absence of cocaine, the quenching of ATMND fluorescence increased as the number of base-pairs between the two CBSA binding domains increased from 5- to 8-bp (ESI, Fig. S5B†). This is probably due to the increased thermo-stability of CBSA assembly, even without target. Upon addition of cocaine, we found a strong correlation between ATMND quenching from CBSA assembly and cocaine concentration in the range of 0 to 50 μM (ESI, Fig. S5C†). Compared to CBSA-5325, quenching saturation occurred at lower target concentrations for the other CBSAs, in keeping with the assumption that the CBSA binding affinity can be enhanced with additional base-pairs. However, considerable background assembly was observed for CBSA-5435 and CBSA-5445 in the absence of cocaine, reducing their target-induced signal gain. We thus found that CBSA-5335 is most responsive and exhibited the most extensive target-induced CBSA assembly, and we therefore used this construct for subsequent sensor development.

**Fig. 4:**
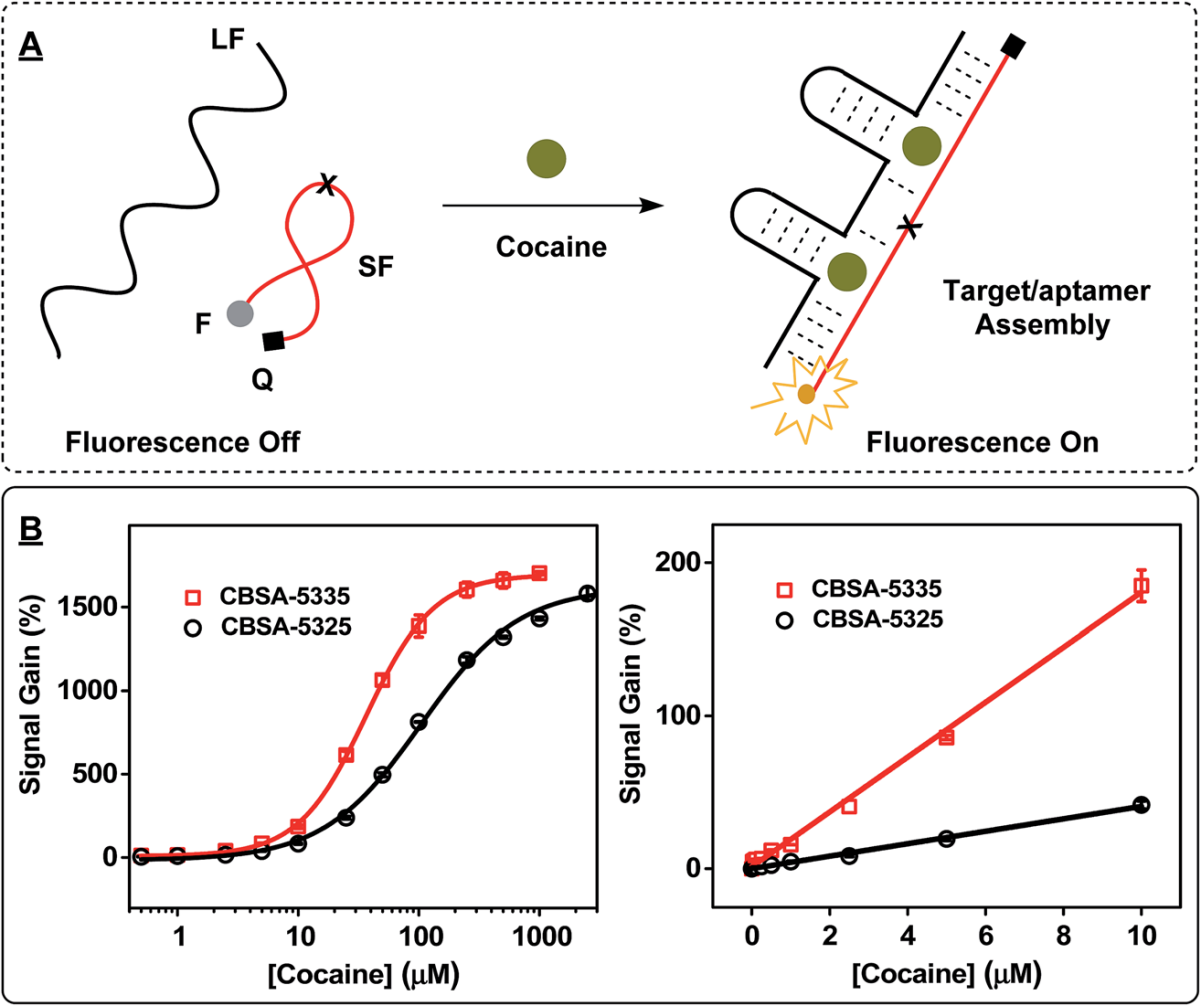
Effect of target-binding affinity (*K*_1/2_) on CBSA cooperativity. (A) The working principle of the CBSA-based fluorophore/quencher assay. (B) Calibration curves for the assay at cocaine concentrations ranging from 0 to 2500 μM (left). Right panel shows a linear response at 0–10 μM. Reactions were performed with 1 μM CBSA long fragment, 1 μM fluorophore/quencher-modified CBSA short fragment, and different concentrations of cocaine in 10 mM Tris–HCl, 100 μM MgCl_2_ (pH 7.4) at room temperature.

We then produced fluorophore/quencher-modified derivatives of CBSA-5325 and CBSA-5335 to achieve sensitive detection of cocaine. The short fragment was modified with an IowaBlack RQ quencher at its 5′ terminus and a Cy5 fluorophore at its 3′ terminus. In the absence of cocaine, the two CBSA fragments remain separate, and the flexibility of the unbound short fragment routinely brings the fluorophore into close proximity with the quencher, resulting in very low fluorescence (Fig. 4A, left). In the presence of cocaine, the two fragments assemble to form a rigid target/aptamer structure that separates the fluorophore/quencher pair, producing increased fluorescence (Fig. 4A, right). We used these two fluorophore/quencher-modified CBSAs to generate calibration curves for cocaine concentrations ranging from 0–1000 μM (Fig. 4B; spectra of CBSA-5335 shown in ESI, Fig. S6†) and used the Hill equation (eqn (1)) to fit the binding curve to calculate *K*_1/2_ and the Hill coefficient (*n*_H_):^30,38^
1
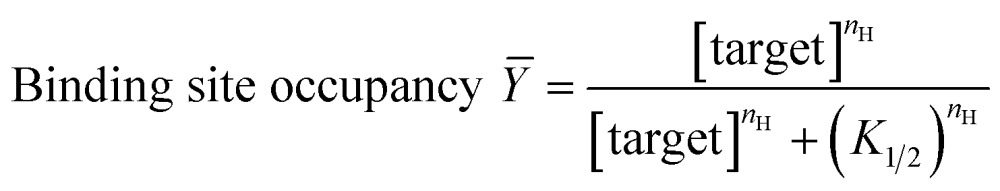



We determined a *K*_1/2_ of 106 μM with an *n*_H_ of 1.1 for CBSA-5325 and a *K*_1/2_ of 36 μM with an *n*_H_ of 1.5 for CBSA-5335 (where *K*_1/2_ represents the cocaine concentration at which half of the binding domains are occupied and *n*_H_ describes the order of binding cooperativity).^38^ An *n*_H_ of 1.5 clearly indicates higher cooperativity between the two binding domains of CBSA-5335. We found that the measurable LOD was 500 nM (4.5 ± 0.8%) and 50 nM (4.3 ± 0.9%) for CBSA-5325 and CBSA-5335, respectively (Fig. 4B). Clearly, the target-binding affinity of CBSA can be affected by the cooperativity of the two binding domains. Note that CBSA-5335 has the highest cocaine affinity of any split aptamer described to date, and the LOD of CBSA-5335 is more than 200-fold lower than that of a previously-described single-domain, split aptamer-based cocaine assay using a similar sensing platform (LOD = 10 μM).^17^

We further used ITC to characterize the binding mechanism and affinity of CBSA-5335 for its target at both sites. Recognizing that the *K*_D_ values of the aptamers were in the μM range, we set up a titration with a high cocaine : aptamer ratio to obtain accurate *K*_D_ values for each binding scenario (the adjacent binding pocket empty, or occupied by cocaine), where *K*_D_ represents the ligand concentration at which half of the receptor sites are occupied at equilibrium.^39^ The resulting two-phase titration curve confirmed the interaction of cocaine with the two binding domains of CBSA-5335 (ESI, Fig. S7A†). The binding stoichiometry between cocaine and CBSA was manually set as two, because previous studies have demonstrated that one cocaine-binding aptamer binds a single molecule of cocaine.^31^ ITC data of CBSA-5335 were then fitted with both independent-sites and cooperative-sites models^40^ with two binding sites (ESI, Fig. S7A,† black broken line represented independent-sites model and red solid line represented cooperative-sites model). We observed better fitting using the cooperative-sites model, and determined that the *K*_D_ for the initial and secondary cocaine-binding events were 116 and 36 μM, respectively (ESI, Fig. S7A†). Based on these *K*_D_s, we calculated a *K*_1/2_ of 65 μM and an *n*_H_ of 1.3 for CBSA-5335,^41^ which is comparable with the results obtained *via* the CBSA-5335-based fluorophore/quencher assay (*K*_1/2_ = 36 μM and *n*_H_ = 1.5). Notably, the low *K*_1/2_ of CBSA-5335 represents a 56-fold higher target binding affinity relative to its single-domain PSA (*K*_D_ = 2 mM, ESI, Fig. S7B†).

**Fig. 5:**
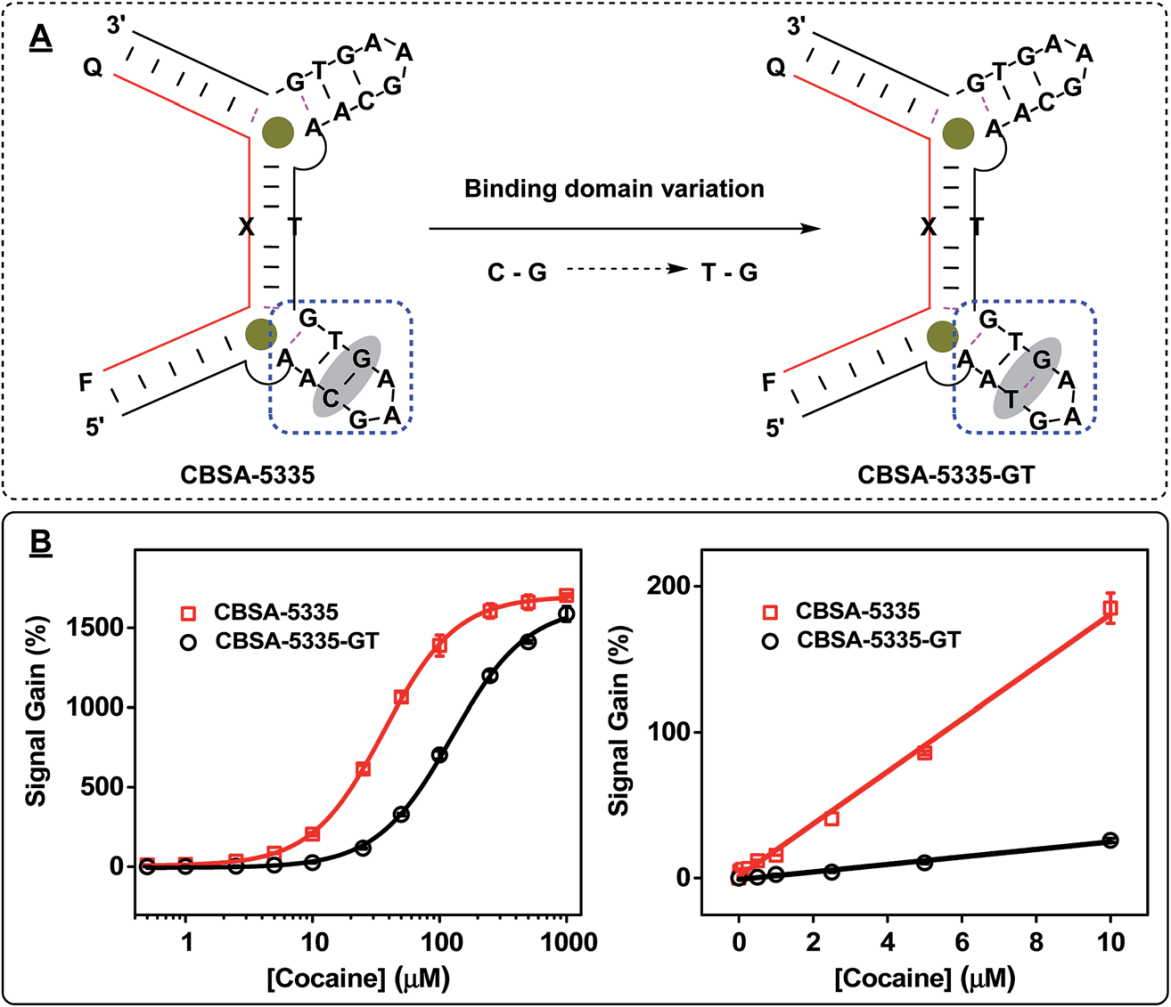
Effect of the intrinsic affinity of the PSA on CBSA target-binding affinity (*K*_1/2_). (A) We generated CBSA-5335-GT by replacing a G–C base-pair (highlighted) in the 5′ binding domain of CBSA-5335 with a G–T wobble pair. (B) Calibration curves for the assay at cocaine concentrations ranging from 0 to 1000 μM after a 15 min incubation (left), with a linear response at 0–10 μM (right). Experiments were performed with 1 μM CBSA long fragment, 1 μM fluorophore/quencher-modified CBSA short fragment, and different concentrations of cocaine in 10 mM Tris–HCl, 100 μM MgCl_2_ (pH 7.4) at room temperature.

We believed that the target-binding affinity of CBSA could be affected by the intrinsic binding affinity of its parent split aptamer. To address this point, we replaced a G–C pair in the 5′ binding domain of CBSA-5335 with a wobble G–T pair to form CBSA-5335-GT. This alteration in stem 3 of the cocaine-binding aptamer reduces its binding affinity to cocaine^31^ (Fig. 5A), but we expected the resulting CBSA to still retain its cooperativity. Indeed, our fluorescence results demonstrated that both CBSAs have identical cooperativity (*n*_H_ = 1.5) (Fig. 5B). However, since the intrinsic affinity of 38-GC is 4-fold higher than the parent split aptamer variant used for CBSA-5335-GT, CBSA-5335 yields a lower *K*_1/2_ (33 μM) and better sensitivity (LOD = 50 nM in buffer) than CBSA-5335-GT (*K*_1/2_ = 125 μM and LOD = 500 nM in buffer) (Fig. 5B).

We subsequently confirmed that our CBSA-5335-based fluorophore/quencher assay is capable of equally sensitive cocaine detection in saliva samples. The excitation wavelength for Cy5 (648 nm) does not induce auto-fluorescence in the saliva matrix, and thus produces minimal background fluorescence (ESI, Fig. S8†). Additionally, the high quenching efficiency of the IowaBlack RQ quencher^42^ allowed robust detection of cocaine at very low concentrations. To ensure that our CBSA-5335-based fluorophore/quencher assay can be reliably used in real-world (*i.e.*, clinical or field) settings, we evaluated the assay's performance according to Scientific Working Group for Forensic Toxicology (SWGTOX) Standard Practices.^43^ To this end, we performed detailed experiments to investigate matrix effects, reaction time, limit of detection, interference effects and bias and precision in saliva samples.

**Fig. 6:**
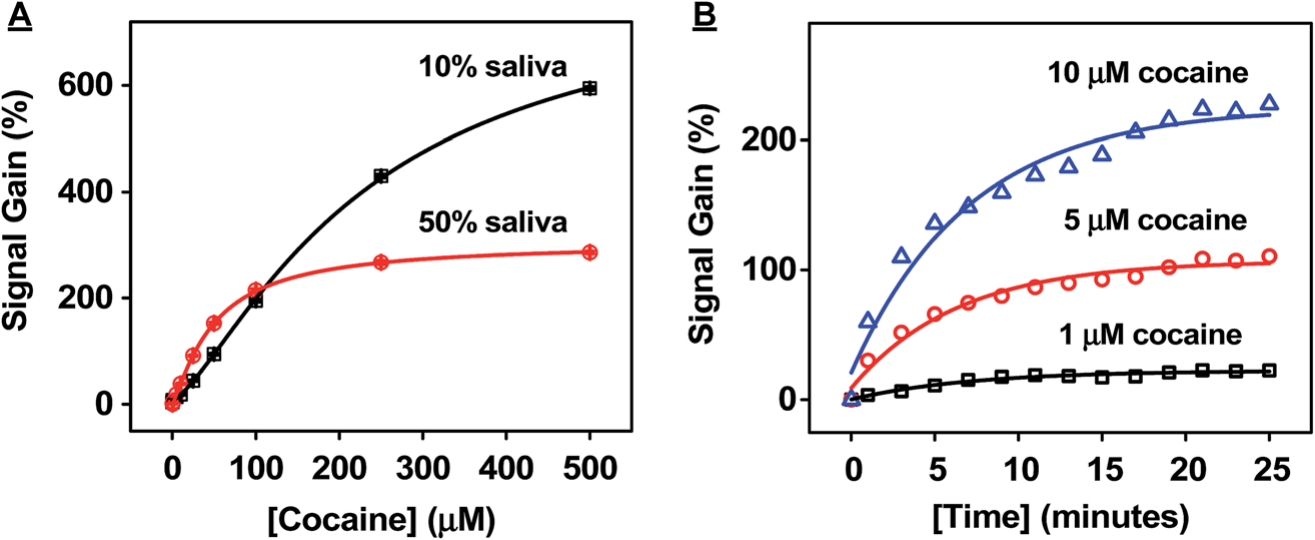
Successful detection of cocaine in saliva samples with fluorophore/quencher-modified CBSA-5335. (A) Dilution effects on cocaine detection in saliva. Saliva samples spiked with cocaine were tested with CBSA-5335 at 50% or 10% dilutions. Calibration curves were constructed based on signal gain at each concentration of cocaine in the pre-dilution samples. (B) Time course of CBSA-5335 in the presence of 1, 5 and 10 μM cocaine in 10% pooled samples.

To test the matrix effects on assay performance, we mixed eight different saliva samples collected from healthy and drug-free donors of diverse gender and ethnic backgrounds as a pooled matrix. The pooled matrix was then spiked with different concentrations of cocaine (0 to 500 μM) and diluted with binding buffer 1 : 1 (50%) or 1 : 9 (10%) before being applied to the CBSA-5335-based fluorophore/quencher assay. Our results showed that the 10% dilution resulted in a higher signal gain with a broader dynamic range (0–100 μM in the initial saliva sample) compared to the 50% dilution (0–25 μM in the initial sample) (Fig. 6A). Additionally, the 10% dilution compensates for variations (such as salt concentration and pH) in individual saliva samples, and we therefore used 10% dilutions for subsequent experiments.

We also monitored the time course of our CBSA-5335-based fluorophore/quencher assay in the 10% pooled saliva matrix. We found that the fluorescence signal greatly increased with the increase of reaction time upon the addition of 1, 5 or 10 μM cocaine and 85% of the maximum signal was obtained after 15 min (Fig. 6B). Clearly, the fast reaction time of our assay is suitable for on-site detection due to the rapid assembly of the cocaine–CBSA complex in saliva samples.

**Fig. 7:**
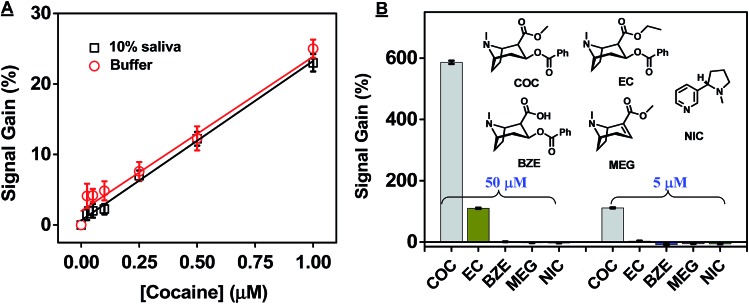
High sensitivity and specificity of our CBSA-5335-based fluorophore/quencher assay for cocaine detection in saliva. (A) Calibration curve for the assay in buffer and 10% saliva. (B) Signal gains from the CBSA assay in the presence of 50 μM (left) and 5 μM (right) cocaine (COC) or potential interferents including cocaethylene (EC), benzoylecgonine (BZE), anhydroecgonine methyl ester (MEG) and nicotine (NIC) in 10% saliva. Inset shows the interferent structures. Error bars show standard deviation of signal gain from three measurements at each concentration.

The CBSA-5335-based fluorophore/quencher assay also demonstrates excellent performance in target detection in complex samples. Studies have shown that cocaine concentrations are generally higher in saliva than serum within the first few hours of administration,^44,45^ and the European Union's Driving Under the Influence of Drugs, Alcohol and Medicines (DRUID) program identified 510 nM as the recommended cut-off sensitivity for road-side screening of cocaine in undiluted saliva.^44^ To determine the sensitivity of our assay, we generated a calibration curve in 10% saliva samples, obtaining a linear range from 0 to 10 μM and a measurable LOD of 50 nM (signal gain 3.3 ± 0.8%, Fig. 7A and ESI, Fig. S9†). This suggests that, accounting for the ten-fold sample dilution, our assay can meet the recommendations established by DRUID for on-site detection of cocaine, with a detectable LOD equivalent to 500 nM in undiluted saliva. In contrast, split aptamers containing a single binding domain have previously achieved LODs of 30 nM in 0.5% saliva,^46^ 5 μM,^47^ and 3.8 μM^48^ in 25% saliva (ESI, Table S1†). Two factors contribute to the high sensitivity of our CBSA assay. First, the low thermo-stability of the split aptamer greatly suppresses non-specific assembly of the CBSA fragments. Second, the cooperative binding from the two target-binding domains significantly increases the CBSA's affinity.

We then investigated the specificity of the CBSA-5335-based fluorophore/quencher assay for cocaine *versus* structurally-similar and -dissimilar interferents in saliva. Benzoylecgonine (BZE), anhydroecgonine methyl ester (MEG), and cocaethylene (EC) are major structurally-similar metabolites of cocaine that are secreted into oral fluids.^49^ We tested our CBSA-5335-based assay with high concentrations of these metabolites as well as nicotine (NIC), since tobacco is widely used among cocaine users. We found that our CBSA assay showed excellent cocaine specificity: our results demonstrated no measurable signal from 50 μM of BZE, MEG, or NIC and only 19% and 3% cross-reactivity to 50 μM and 5 μM EC, respectively, in 10% saliva (Fig. 7B). The results are consistent with previously reported assays^17,20^ based on single-domain cocaine-binding split aptamers, demonstrating that CBSA retains excellent specificity for its target molecule in saliva. Finally, we tested the bias and precision of our CBSA assay by spiking cocaine at low, medium, and high concentrations into 10% saliva samples (final concentration of 1, 5 or 10 μM) from eight different individuals (ESI, Fig. S10A†). Using the pooled saliva as a standard, the average bias of signal gain obtained in these individual samples was 12.7%, –0.4% and –5.8% for 1, 5 and 10 μM cocaine, respectively. At 1, 5 and 10 μM, the coefficients of variation (CV) within samples were 7.1%, 5.2% and 9.0%, respectively, and the CV between runs were 7.3%, 5.1% and 8.5%, respectively (ESI, Fig. S10B†). Thus, the bias and CV were consistently below the acceptable cut-off (20%) for drug-screening methods,^43^ further demonstrating the immediate feasibility of our CBSA-based assay for on-site drug screening.

## Conclusions

We have demonstrated greatly improved split-aptamer-based sensing of cocaine in both buffer and saliva with a CBSA that incorporates two tandem target-binding domains into a single split aptamer. The CBSA fragments remain separate in the absence of target but specifically assemble upon addition of cocaine, and show high sensitivity to target concentration as a result of increased binding affinity caused by cooperative binding behavior. We achieved rapid, one-step detection of cocaine by simply mixing our CBSA components, which consist of a fluorophore/quencher-modified short fragment and an unmodified long fragment, with a cocaine-containing sample at room temperature. A measurable LOD of 50 nM in buffer was achieved within 15 minutes without any signal amplification. The sensor's performance was also very robust in saliva, achieving a LOD of 50 nM cocaine in 10% diluted saliva. Our assay also demonstrates very high target specificity, as evidenced by acceptably low cross-reactivity to common interferents, strong reproducibility, as well as a cost of $1.28 per test, confirming the potential value of our CBSA-based assay for on-site detection of cocaine.

Our CBSA-based assay can be generalized in terms of both targets and sensor platforms. Stojanovic *et al.* recently isolated new aptamers for four different steroids *via* heterogeneous SELEX using partially randomized DNA libraries with a pre-designed, three-way-junction binding domain.^50^ We believe that such a selection strategy could similarly be applied for the isolation of aptamers for other drugs of abuse as well as clinically relevant targets such as small-molecule biomarkers, toxins and therapeutics. Based on Heemstra's general approach to split three-way-junction structured aptamers^51^ and the simplicity of engineering a CBSA from a split aptamer, it should be straightforward to develop other CBSA-based assays from the isolated aptamers for which the target-binding domain has been identified. Since many optical and electrochemical sensing strategies have been developed that employ target-induced split aptamer assembly,^21,52–55^ it should be feasible to integrate our CBSA into these platforms. We also foresee the potential to improve the performance of our CBSA-based assay by employing signal amplification techniques^53–57^ that could further enhance its sensitivity.

## Experimental

### Materials

2-Amino-5,6,7-trimethyl-1,8-naphthyridine (ATMND) was purchased from Ryan Scientific and dissolved in dimethyl sulfoxide (DMSO) as a stock solution (50 μM). Cocaine hydrochloride was purchased from Sigma-Aldrich. Benzoylecgonine tetrahydrate, (–)-nicotine, anhydroecgonine methyl ester and cocaethylene were purchased from Cerilliant Corporation and prepared as 50 mM stock solutions in deionized water and stored at 4 °C. Saliva samples were collected from eight healthy and drug-free volunteers (four males and four females) and centrifuged at 20 000 rcf for 15 min, with supernatants stored at 4 °C before use. The pooled saliva matrices were prepared by mixing 1 mL of saliva supernatant from each donor. All DNA molecules were ordered from Integrated DNA Technologies and purified with HPLC and the sequences are listed in the ESI (Table S2†). DNA was dissolved in PCR grade water and DNA concentrations were measured with a NanoDrop 2000 (Thermo Scientific).

### Characterization of split aptamer assembly using ATMND

For each ATMND-binding assay, we mixed 10 μL of 10× binding buffer (100 mM Tris, 1 mM MgCl_2_, pH 7.4), 85 μL of deionized water, 1 μL of each split aptamer fragment (final concentration 1 μM), 1 μL ATMND solution (final concentration 200 nM) and 2 μL of cocaine at different concentrations into wells of a 96-well plate. Fluorescence intensity was measured using Tecan M1000Pro with excitation at 358 nm and emission at 405 nm at a 2 minute time interval until the fluorescence intensity was stable. Each sample was analyzed in triplicate, and the means and standard deviations were plotted. Quenching was calculated by (*F*_A_ – *F*)/*F*_A_ × 100%, where *F*_A_ is the fluorescence of 200 nM ATMND in 1× binding buffer alone and *F* is the fluorescence of the ATMND–CBSA mixture with or without cocaine. Error bars show standard deviations obtained from three measurements.

### Isothermal titration calorimetry (ITC) experiments

ITC experiments were performed with a MicroCal ITC200 (GE Healthcare). Cocaine and split aptamers were prepared with 1× binding buffer. The sample cell was initially loaded with 20 μM of CBSA-5335 or PSA. 4 mM of cocaine titrant was loaded into the syringe. Each experiment typically consisted of 39 successive 1 μL injections after a 0.4 μL purge injection with spacing of 210 seconds to a final molar ratio of 43 : 1 (cocaine : aptamer). Split-aptamer experiments were performed at 20 °C. The raw data were first corrected based on the heat of dilution of cocaine, and then analyzed with the MicroCal analysis kit integrated into Origin 7 software. The titration curve of PSA was fitted with a single-site binding model and the titration curve of CBSA-5335 was fitted with independent-sites and cooperative-sites models with two binding sites.

### Fluorophore/quencher-modified CBSA-5325, -5335, or -5335-GT for detection of cocaine

For each fluorophore/quencher-modified CBSA-based assay, we mixed 10 μL of 10× binding buffer, 83 μL of deionized water, 1 μL of the CBSA long fragment and 1 μL of the fluorophore/quencher-modified CBSA short fragment (final concentration 1 μM each), and 5 μL of cocaine at different concentrations into wells of a 96-well plate. We measured the fluorescence intensity with a Tecan M1000Pro with excitation at 648 nm and emission at 668 nm at room temperature after 15 min of incubation. Each sample was analyzed in triplicate, and the means and standard deviations were plotted. The data were fitted with the Hill equation using Origin 9 software to calculate the Hill coefficient (*n*_H_) and cocaine concentration producing half occupancy (*K*_1/2_). The signal gain was calculated by (*F* – *F*_0_)/*F*_0_ × 100%, where *F*_0_ is the fluorescence of the CBSA mixture without cocaine and *F* is the fluorescence of CBSA mixtures with different concentrations of cocaine. Error bars show standard deviation of signal gains from three individual measurements at each cocaine concentration.

### Assessing saliva matrix and dilution effects on the CBSA-5335-based fluorophore/quencher assay

Cocaine was first spiked into the pooled saliva (FIU IRB: IRB-13-0320) to create artificial samples with concentrations ranging from 0.25 to 500 μM. Each sample was prepared by mixing 10 μL of 10× binding buffer, 1 μL of CBSA long fragment and 1 μL of fluorophore/quencher-modified CBSA short fragment (final concentration 1 μM each) into wells of a 96-well plate. 10 or 50 μL of sample for each cocaine concentration was added into the well to analyze the 10% or 50% saliva matrices and deionized water was added to bring each well's volume to 100 μL. Fluorescence intensity with excitation at 648 nm and emission from 655–850 nm was scanned with a Tecan M1000Pro at room temperature after 15 min of incubation. Each sample was analyzed in triplicate, and the mean and standard deviation of the signal gain at different cocaine concentrations were plotted.

### Determining the sensitivity and dynamic range of CBSA-5335-based fluorophore/quencher assay in saliva

For cocaine detection in 10% saliva, we combined 5 μL of cocaine in solutions of concentrations ranging from 0.001 to 10 μM with 10 μL of pooled saliva, 10 μL of 10× binding buffer, 1 μL of CBSA long fragment, 1 μL of fluorophore/quencher-modified CBSA short fragment (final concentration 1 μM each), and 73 μL of deionized water into wells of a 96-well plate. Fluorescence intensity with excitation at 648 nm and emission at 668 nm was measured with a Tecan M1000Pro at room temperature after 15 min of incubation. Each sample was analyzed in triplicate, and the mean and standard deviation of the signal gain at different cocaine concentrations were plotted. A control calibration curve in buffer was also generated with cocaine concentrations ranging from 0.001 to 10 μM as described above. The signal gain was calculated by (*F* – *F*_0_)/*F*_0_ × 100%, where *F*_0_ is the fluorescence without cocaine and *F* is the fluorescence with different concentrations of cocaine. Error bars show standard deviations from three measurements. The measurable LOD was determined using the lowest non-zero calibrator concentration according to the Scientific Working Group for Forensic Toxicology (SWGTOX) Standard Practices for Method Validation in Forensic Toxicology.^43^ In all cases, the calibrators (cocaine standard concentration) were analyzed over three runs, and the lowest concentration achieving mean signal gain higher than 3.3 times the standard deviation was defined as the measurable LOD.

### Determining the target specificity of CBSA-5335-based fluorophore/quencher assay in saliva

The CBSA-5335 fluorophore/quencher assay was performed as described above with cocaine, cocaethylene, benzoylecgonine, anhydroecgonine methyl ester or nicotine at concentrations of 5 or 50 μM in 10% saliva. Each sample was analyzed in triplicate and the mean and standard deviation of the signal gain at different cocaine concentrations were plotted. The cross-reactivity of each analyte at each concentration was calculated as a percentage based on Sig_ANA_/Sig_COC_ × 100%, where Sig_ANA_ is the signal gain achieved by a given interferent and Sig_COC_ is the signal gain achieved by cocaine at the same concentration.

### Determining the precision and bias of CBSA-5335-based fluorophore/quencher assay in saliva

The CBSA-5335 fluorophore/quencher assay was performed as described above in 10% diluted saliva matrices collected from eight different donors, 10% diluted pooled saliva matrices and binding buffer. We performed six measurements of samples containing final cocaine concentrations of 0, 1, 5 and 10 μM, and plotted the mean and standard deviation of the signal gain at different cocaine concentrations. The bias of each cocaine concentration was calculated as (mean_sam_ – mean_pool_)/mean_pool_ × 100%, where mean_sam_ is the mean signal gain obtained in 10% saliva matrices collected from different donors, and mean_pool_ is the mean signal gain obtained in the 10% pooled saliva matrix. The precision within samples or between runs at different cocaine concentrations was calculated by performing a one-way ANOVA test with the measurement number (6) as the grouping variable. Within-sample precision at each cocaine level was calculated as 
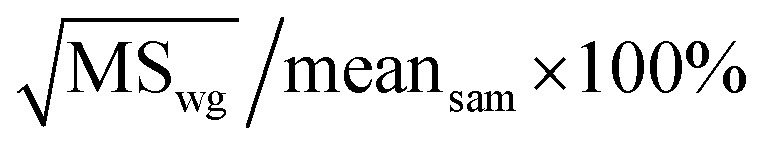
, where MS_wg_ is the within-group mean square obtained from the ANOVA table, and mean_sam_ is the mean of signal gains obtained in 10% saliva matrices collected from different donors. Between-run precision at each cocaine level was calculated as 
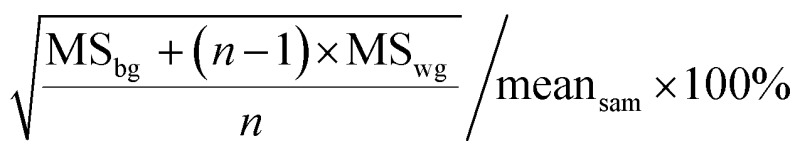
, where MS_bg_ is the between-group mean square obtained from the ANOVA table, and *n* is the total number of measurements (*n* = 6).

## Conflict of interest

The authors declare no competing financial interest.

## References

[cit1] Ellington A. D., Szostak J. W. (1990). Nature.

[cit2] Tuerk C., Gold L. (1990). Science.

[cit3] Lee J. F., Hesselberth J. R., Meyers L. A., Ellington A. D. (2004). Nucleic Acids Res..

[cit4] Ruigrok V. J. B., Levisson M., Eppink M. H. M., Smidt H., van der Oost J. (2011). Biochem. J..

[cit5] Jayasena S. D. (1999). Clin. Chem..

[cit6] Mok W., Li Y. F. (2008). Sensors.

[cit7] Stojanovic M. N., de Prada P., Landry D. W. (2001). J. Am. Chem. Soc..

[cit8] Yang K. A., Barbu M., Halim M., Pallavi P., Kim B., Kolpashchikov D. M., Pecic S., Taylor S., Worgall T. S., Stojanovic M. N. (2014). Nat. Chem..

[cit9] Bock L. C., Griffin L. C., Latham J. A., Vermaas E. H., Toole J. J. (1992). Nature.

[cit10] Huizenga D. E., Szostak J. W. (1995). Biochemistry.

[cit11] Mairal T., Ozalp V. C., Lozano Sánchez P., Mir M., Katakis I., O'Sullivan C. K. (2008). Anal. Bioanal. Chem..

[cit12] Lee J. H., Yigit M. V., Mazumdar D., Lu Y. (2010). Adv. Drug Delivery Rev..

[cit13] Cho E. J., Lee J. W., Ellington A. D. (2009). Annu. Rev. Anal. Chem..

[cit14] McKeague M., DeRosa M. C. (2012). J. Nucleic Acids.

[cit15] Stojanovic M. N., Landry D. W. (2002). J. Am. Chem. Soc..

[cit16] Baker B. R., Lai R. Y., Wood M. S., Doctor E. H., Heeger A. J., Plaxco K. W. (2006). J. Am. Chem. Soc..

[cit17] Stojanovic M. N., de Prada P., Landry D. W. (2000). J. Am. Chem. Soc..

[cit18] Zou R. X., Lou X. H., Ou H. C., Zhang Y., Wang W. J., Yuan M., Guan M., Luo Z. F., Liu Y. Y. (2012). RSC Adv..

[cit19] Nie J., Deng Y., Deng Q. P., Zhang D. W., Zhou Y. L., Zhang X. X. (2013). Talanta.

[cit20] Sharma A. K., Heemstra J. M. (2011). J. Am. Chem. Soc..

[cit21] Sharma A. K., Kent A. D., Heemstra J. M. (2012). Anal. Chem..

[cit22] Perutz M. F., Wilkinson A. J., Paoli M., Dodson G. G. (1998). Annu. Rev. Biophys. Biomol. Struct..

[cit23] Meyer T., Holowka D., Stryer L. (1988). Science.

[cit24] Krell T., Terán W., Mayorga O. L., Rivas G., Jiménez M., Daniels C., Molina-Henares A. J., Martínez-Bueno M., Gallegos M. T., Ramos J. L. (2007). J. Mol. Biol..

[cit25] Bray D. (1995). Nature.

[cit26] Mandal M., Lee M., Barrick J. E., Weinberg Z., Emilsson G. M., Ruzzo W. L., Breaker R. R. (2004). Science.

[cit27] Sudarsan N., Hammond M. C., Block K. F., Welz R., Barrick J. E., Roth A., Breaker R. R. (2006). Science.

[cit28] Jose A. M., Soukup G. A., Breaker R. R. (2001). Nucleic Acids Res..

[cit29] Simon A. J., Vallée-Bélisle A., Ricci F., Watkins H. M., Plaxco K. W. (2014). Angew. Chem., Int. Ed..

[cit30] Simon A. J., Vallée-Bélisle A., Ricci F., Plaxco K. W. (2014). Proc. Natl. Acad. Sci. U. S. A..

[cit31] Roncancio D., Yu H. X., Xu X. W., Wu S., Liu R., Debord J., Lou X. H., Xiao Y. (2014). Anal. Chem..

[cit32] Sato Y., Kageyama T., Nishizawa S., Teramae N. (2013). Anal. Sci..

[cit33] Sato Y., Nishizawa S., Yoshimoto K., Seino T., Ichihashi T., Morita K., Teramae N. (2009). Nucleic Acids Res..

[cit34] Neves M. A. D., Reinstein O., Saad M., Johnson P. E. (2010). Biophys. Chem..

[cit35] Reinstein O., Yoo M., Han C., Palmo T., Beckham S. A., Wilce M. C. J., Johnson P. E. (2013). Biochemistry.

[cit36] Owczarzy R., Moreira B. G., You Y., Behlke M. A., Walder J. A. (2008). Biochemistry.

[cit37] Neves M. A. D., Reinstein O., Johnson P. E. (2010). Biochemistry.

[cit38] Hill A. (1910). J. Physiol..

[cit39] Turnbull W. B., Daranas A. H. (2003). J. Am. Chem. Soc..

[cit40] Freiburger L. A., Auclair K., Mittermaier A. K. (2009). ChemBioChem.

[cit41] Dahlquist F. W., The meaning of scatchard and hill plots, in Enzyme Structure Part F, Methods in Enzymology, ed. C. H. W. Hirs and S. N. Timasheff, Academic Press, Cambridge, MA, 1978, vol. 48, pp. 270–299. 10.1016/s0076-6879(78)48015-2 345049

[cit42] Johansson M. K. (2006). Methods Mol. Biol..

[cit43] Scientific Working Group for Forensic Toxicology (SWGTOX) (2013). J. Anal. Toxicol..

[cit44] Gjerde H., Langel K., Favretto D., Verstraete A. G. (2014). J. Anal. Toxicol..

[cit45] Schramm W., Craig P. A., Smith R. H., Berger G. E. (1993). Clin. Chem..

[cit46] Ma D. L., Wang M. D., He B. Y., Yang C., Wang W. H., Leung C. H. (2015). ACS Appl. Mater. Interfaces.

[cit47] Shi Y., Dai H. C., Sun Y. J., Hu J. T., Ni P. J., Li Z. (2013). Analyst.

[cit48] Du Y., Chen C. G., Yin J. Y., Li B. L., Zhou M., Dong S. J., Wang E. K. (2010). Anal. Chem..

[cit49] Cone E. J., Hillsgrove M., Darwin W. D. (1994). Clin. Chem..

[cit50] Yang K. A., Pei R. J., Stefanovic D., Stojanovic M. N. (2012). J. Am. Chem. Soc..

[cit51] Kent A. D., Spiropulos N. G., Heemstra J. M. (2013). Anal. Chem..

[cit52] Freeman R., Sharon E., Tel-Vered R., Willner I. (2009). J. Am. Chem. Soc..

[cit53] Lu C. H., Wang F., Willner I. (2012). Chem. Sci..

[cit54] Liu X. Q., Freeman R., Willner I. (2012). Chem.–Eur. J..

[cit55] Li Q., Wang Y. D., Shen G. L., Tang H., Yu R. Q., Jiang J. H. (2015). Chem. Commun..

[cit56] Niu S. Y., Lou X. F., Jiang Y., Lin J. H. (2012). Anal. Lett..

[cit57] Zhu Z., Wu C., Liu H., Zou Y., Zhang X., Kang H., Yang C. J., Tan W. (2010). Angew. Chem., Int. Ed..

