# Supplementary material for: A cooperative-binding split aptamer assay for rapid, specific and ultra-sensitive fluorescence detection of cocaine in saliva
Source: Chem Sci. 2016 Jul 29;8(1):131–41. doi: 10.1039/c6sc01833e (PMC5308383; doi:10.1039/c6sc01833e)
Supplement: Supplementary file 1 [file SC-008-C6SC01833E-s001.pdf]

Electronic Supplementary Material (ESI) for Chemical Science.
This journal is © The Royal Society of Chemistry 2016

Supporting information for the article:

A cooperative-binding split aptamer assay for rapid, specific and ultra-sensitive fluorescence detection of cocaine in saliva

Haixiang Yu,[1] Juan Canoura,[1] Bhargav Guntupalli,[1] Xinhui Lou[2] and Yi Xiao*[1]

[1]Department of Chemistry and Biochemistry, Florida International University, 11200 SW 8th Street, Miami, FL, 33199; [2]Department of Chemistry, Capital Normal University, Xisanhuan North Rd. 105, Beijing, China, 100048.

*Corresponding author: yxiao2@fiu.edu

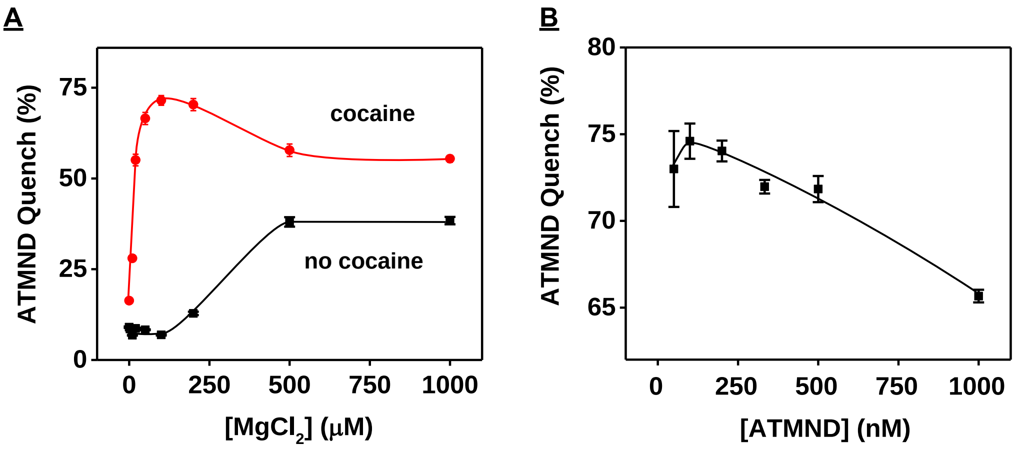

Figure S1: Optimization of Mg^{2+} and ATMND concentrations for our CBSA-based ATMND-binding assay. (A) ATMND (200 nM) quenching by target-induced CBSA assembly upon addition of 250 μM cocaine in the presence of 0-1000 μM Mg^{2+} in 1× binding buffer. Quenching was calculated by $(F_A\text{-}F)/F_A \times 100\%$, where F_A is the fluorescence of 200 nM ATMND in 1× binding buffer alone and F is the fluorescence of the ATMND-CBSA mixture with 250 μM cocaine (red curve) or without cocaine (black curve), respectively. (B) Quenching analysis with ATMND concentrations ranging from 50-1000 nM in 1× binding buffer containing 100 μM Mg^{2+}. Quenching was calculated by $(F_0\text{-}F)/F_0 \times 100\%$, where F_0 and F are the fluorescence of the ATMND-CBSA in the absence and presence of cocaine, respectively.

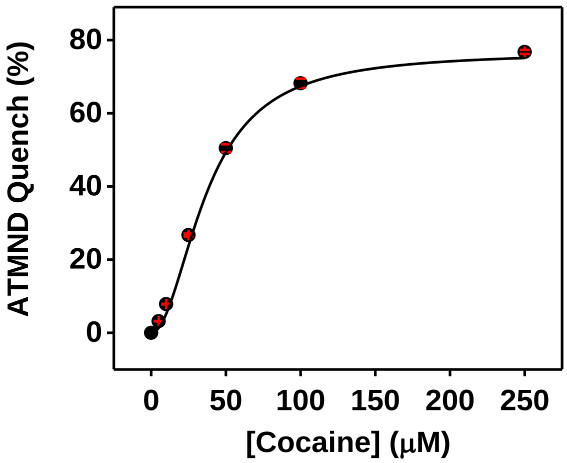

Figure S2: ATMND-reported calibration curve for CBSA-5325 at varying cocaine concentrations. Error bars show standard deviations from three measurements. Quenching was calculated by $(F_0\text{-}F)/F_0 \times 100\%$, where F_0 and F are the fluorescence of the ATMND-CBSA in the absence and presence of cocaine, respectively.

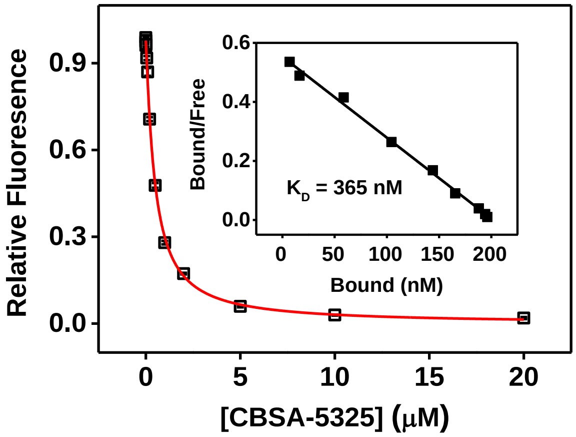

Figure S3: ATMND binding affinity for cocaine-assembled CBSA-5325. ATMND (200 nM) fluorescence was quenched with increasing concentrations of CBSA-5325 in the presence of 1 mM cocaine. Inset: Scatchard plot of the fluorescence data. The K_D was determined based on the negative reciprocal of the slope.

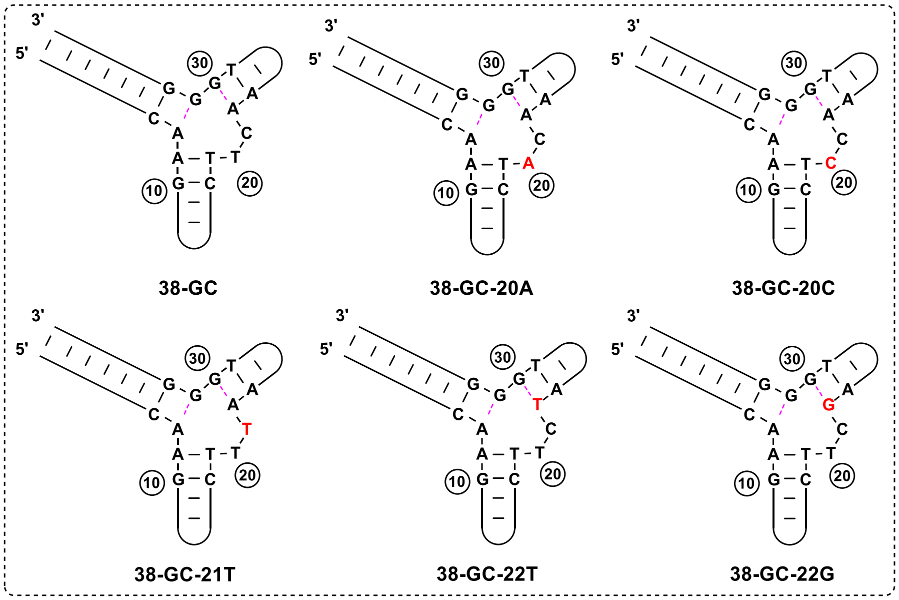

Sequence ID	38-GC	38-GC-20A	38-GC-20C	38-GC-21T	38-GC-22T	38-GC-22G
K_D (μM)	2.60	169	714	49.3	169	>1000

Figure S4: K_D of 38-GC and different 38-GC mutants for cocaine as characterized by ITC. The sample cell was initially loaded with 20 μM of aptamers. 500 μM of cocaine titrant was loaded into the syringe. Each experiment typically consisted of 19 successive 2 μL injections after a 0.4 μL purge injection with spacing of 150 seconds to a final molar ratio of 5.5:1 (cocaine:aptamer). The experiments were performed at 25 °C. The raw data were first corrected based on the heat of dilution of cocaine, and then analyzed with the MicroCal analysis kit integrated into Origin 7 software with a single-site binding model.

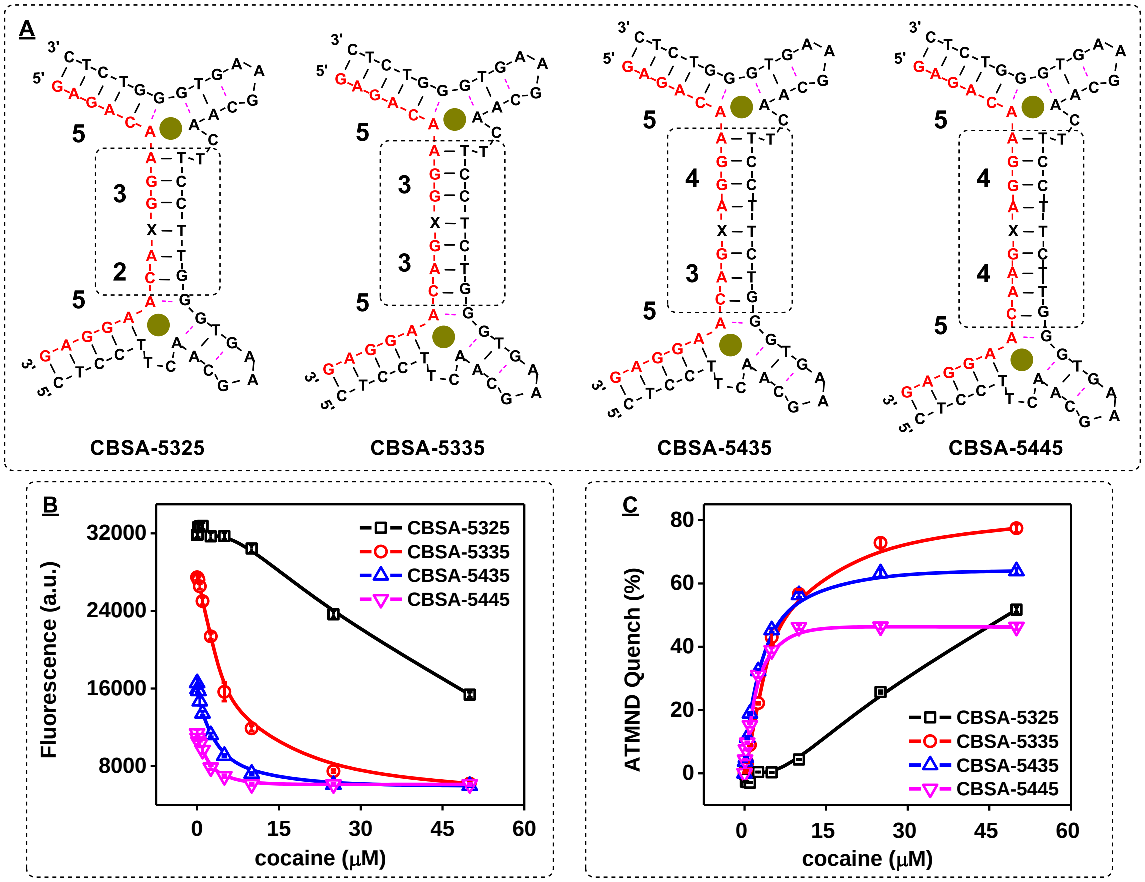

Figure S5: Stem length effects on cocaine-induced CBSA assembly. (**A**) Sequences of CBSA-5325, -5335, -5435 and -5445. (**B**) ATMND (200 nM) fluorescence upon addition of 1 μM CBSA-5325, -5335, -5435 or -5445 at various cocaine concentrations (0 - 50 μM). (**C**) ATMND-reported calibration curve for these CBSA variants at varying cocaine concentrations. Quenching was calculated by $(F_0-F)/F_0\times100\%$, where F_0 and F are the fluorescence of the ATMND-CBSA in the absence and presence of cocaine, respectively.

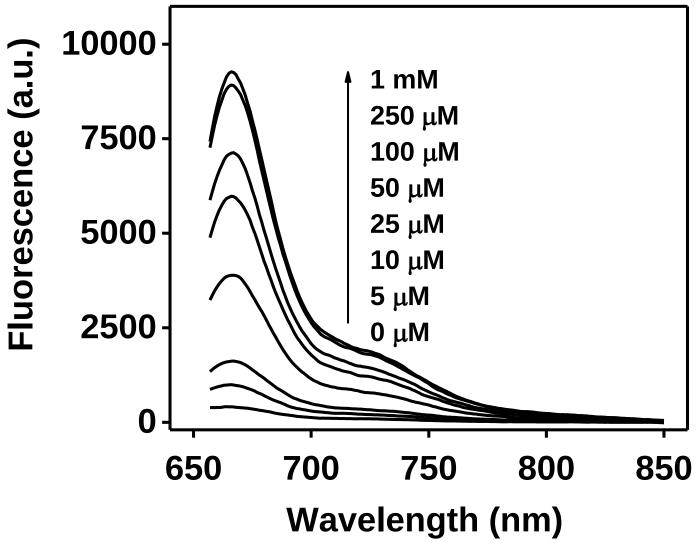

Figure S6: Spectra of CBSA-5335-based fluorescent detection of cocaine in 1× binding buffer. In the absence of cocaine, we observed negligible emission from 655 to 850 nm when excited at 648 nm. The fluorescence peak at 668 nm increased with increasing cocaine concentration.

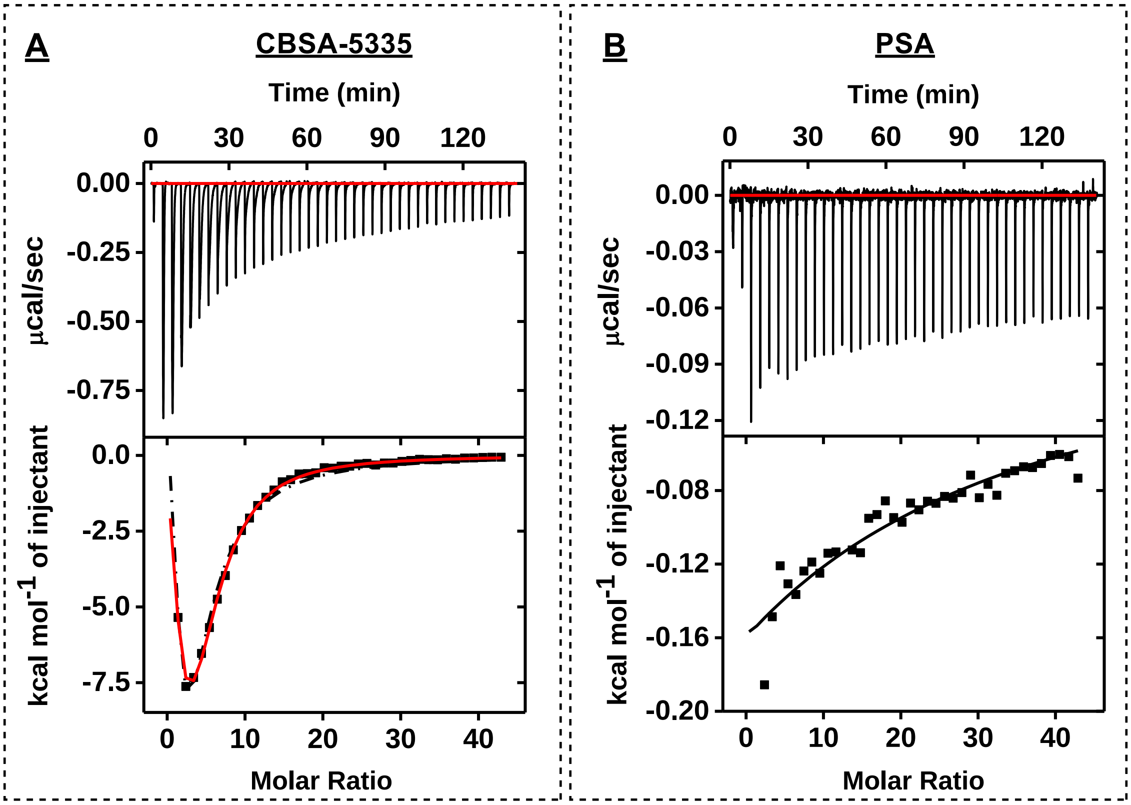

Figure S7: Characterization of cocaine binding affinity of CBSA-5335 and PSA using ITC. Top panels present raw data showing the heat generated from each titration of cocaine for (**A**) CBSA-5335 (black broken line represented independent-sites model and red solid line represented cooperative-sites model) and (**B**) PSA. Bottom panels show the integrated heat of each titration after correcting for dilution heat of the titrant.

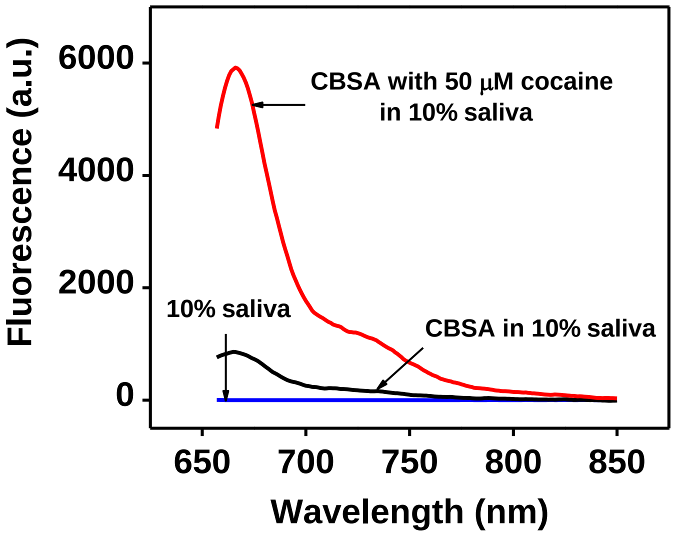

Figure S8: Fluorescent detection of cocaine in saliva with our fluorophore/quencher modified CBSA-5335. 10% saliva (blue) exhibited no emission from 655 to 850 nm when excited at 648 nm. CBSA-5335 alone (black) yielded only slight fluorescence background when excited at 648 nm, but we observed a significant increase in fluorescence at 668 nm with the addition of 50 µM cocaine (red).

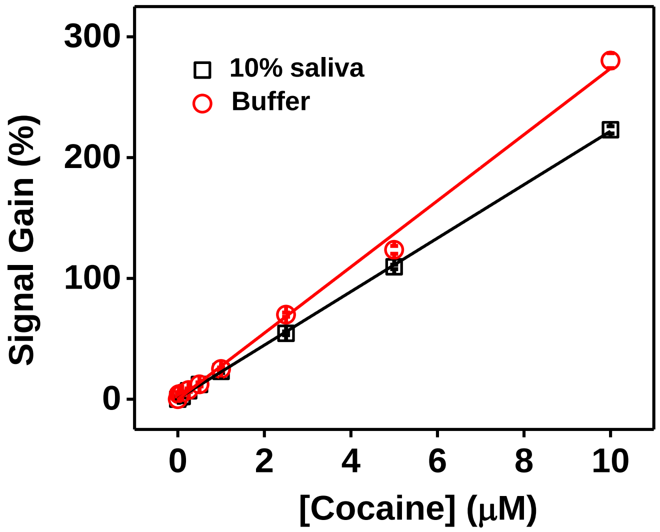

Figure S9: Calibration curve of our CBSA-5335-based fluorophore/quencher assay in 1× binding buffer and 10% saliva at cocaine concentrations ranging from 0 to 10 µM. The signal gain was calculated by $(F-F_0)/F_0\times100\%$, where F_0 is the fluorescence without cocaine and F is the fluorescence with different concentrations of cocaine. Error bars show standard deviation of signal gain from three measurements at each concentration.

A

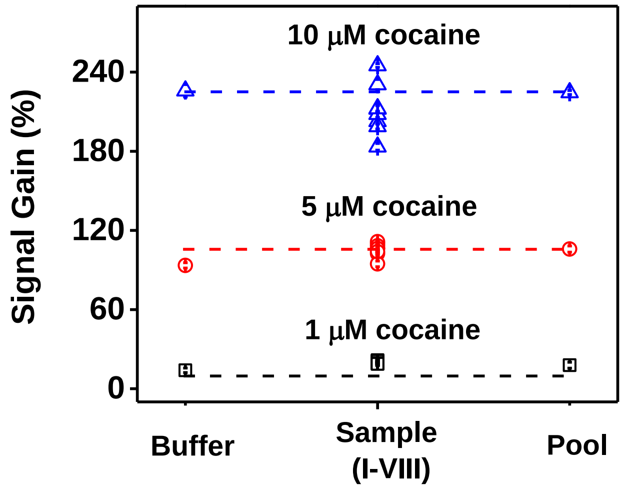

B

Cocaine	Signal gain (%)		Bias	Precision	
Conc. (µM)	Mean (samples)	Mean (pool)	Bias (%)	CV (%) within-sample	CV (%) between-run
1	20	18	12.7	7.1	7.3
5	105	106	-0.4	5.2	5.1
10	212	225	-5.8	9.0	8.5

Figure S10: Bias and precision of the CBSA-5335-based fluorophore/quencher assay. (A) Signal gain obtained from various cocaine concentrations in buffer, 10% saliva collected from different donors (Sample I-VIII) and 10% pooled saliva. Signal gain was calculated by $(F-F_0)/F_0 \times 100\%$, where F_0 is the fluorescence of the CBSA without cocaine and F is the fluorescence of the CBSA with cocaine. Error bars show standard deviations from six measurements. (B) Bias at each cocaine concentration was calculated from the mean signal gain obtained with samples I-VIII and the pooled sample. Within-sample and between-run precision at each cocaine concentration was calculated by one-way ANOVA.

Table S1. Comparison of amplification-free split-aptamer assays for cocaine detection

Signal output	Types of biosample	Dynamic range	Detection time	Reference
Absorbance	1% filtered serum/urine	100 μM	30 min	1
Gel electrophoresis	20% fetal bovine serum	10 μM – 1 mM	8 hr	2
Electrochemical impedance	25% human serum	0.1 μM – 10 μM	30 min	3
Electrochemistry	25% serum/saliva/urine	3.8 μM	40 min	4
Electrochemistry	50% fetal bovine serum	1 mM	5 min	5
Luminescence	0.5% human saliva/urine	30 nM – 300 nM	40 min	6
Fluorescence	25% serum/saliva/urine	5 μM	60 min	7
Fluorescence	10% fetal bovine serum	100 μM	30 min	8
Fluorescence	10% human saliva	50 nM – 10 μM	15 min	this work

Table S2. Sequence ID and DNA sequences used in this work.

Sequence ID	Sequence
**L-PSA**	5'CTCCTTCAACGAAGTGGGTTCC3'
**S-PSA**	5'GG/iSpC3/ACAAGGAG3'
**L-LSA**	5'CTCCTTCAACGAAGTGGGTTCCTTGTCTC3'
**L-5325**	5'CTCCTTCAACGAAGTGGGTTCCTTCAACGAAGTGGGTCTC3'
**S-5325**	5'GAGACAAGG/iSpC3/ACAAGGAG3'
**S-5325-Cy5**	5' /5IAbRQ/ GAGACAAGG/iSpC3/ACAAGGAGT/3Cy5Sp/3'
**L-CBSA-M1**	5'CTCCTTCAACGAAGTGGGTTCCTTCGACGAAGTGGGTCTC3'
**L-CBSA-M2**	5'CTCCTTCGACGAAGTGGGTTCCTTCAACGAAGTGGGTCTC3'
**L-5335**	5'CTCCTTCAACGAAGTGGGTCTCCTTCAACGAAGTGGGTCTC3'
**S-5335**	5'GAGACAAGG/iSpC3/GACAAGGAG3'
**S-5335-Cy5**	5' /5IAbRQ/ GAGACAAGG/iSpC3/GACAAGGAGT/3Cy5Sp/3'
**L-5335-GT**	5'CTCCTTCAATGAAGTGGGTCTCCTTCAACGAAGTGGGTCTC3'
**L-5435**	5'CTCCTTCAACGAAGTGGGTCTTCCTTCAACGAAGTGGGTCTC3'
**S-5435**	5'GAGACAAGGA/iSpC3/GACAAGGAG3'
**L-5445**	5'CTCCTTCAACGAAGTGGGTTCTTCCTTCAACGAAGTGGGTCTC3'
**S-5445**	5'GAGACAAGGA/iSpC3/GAACAAGGAG3'
**38-GC**	5'GGGAGACAAGGAAAATCCTTCAACGAAGTGGGTCTCCC3'
**38-GC-20A**	5'GGGAGACAAGGAAAATCCTACAACGAAGTGGGTCTCCC3'
**38-GC-20C**	5'GGGAGACAAGGAAAATCCTCCAACGAAGTGGGTCTCCC3'
**38-GC-21T**	5'GGGAGACAAGGAAAATCCTTTAACGAAGTGGGTCTCCC3'
**38-GC-22T**	5'GGGAGACAAGGAAAATCCTTCTACGAAGTGGGTCTCCC3'
**38-GC-22G**	5'GGGAGACAAGGAAAATCCTTCGACGAAGTGGGTCTCCC3'

a. /5IAbRQ/ represents Iowa Black RQ b. /iSpC3/ represents internal C3 spacer
c. S-5325 served as short fragment for CBSA-5325, LSA, CBSA-M1 and CBSA-M2
d. /3Cy5Sp/ represents Cy5 e. Mutations are marked in red

References:

1 R. X. Zou, X. H. Lou, H. C. Ou, Y. Zhang, W. J. Wang, M. Yuan, M. Guan, Z. F. Luo and Y. Liu, *RSC Adv.*, 2012, 2, 4636–4638.

2 A. K. Sharma and J. M. Heemstra, *J. Am. Chem. Soc.*, 2011, 133, 12426–12429.

3 D. W. Zhang, F. T. Zhang, Y. R. Cui, Q. P. Deng, S. Krause, Y. L. Zhou and X. X. Zhang, *Talanta*, 2012, 92, 65–71.

4 Y. Du, C. G. Chen, J. Y. Yin, B. L. Li, M. Zhou, S. J.Dong and E. K.Wang, *Anal. Chem.*, 2010, 82, 1556–1563.

5 X. Zuo, Y. Xiao and K. W. Plaxco, *J. Am. Chem. Soc.*, 2009, 131, 6944–6945.

6 D. L. Ma, M. Wang, B. He, C. Yang, W. Wang and C. H.Leung, *ACS Appl. Mater. Interfaces*, 2015, 7, 19060–19067.

7 Y. Shi, H. C. Dai, Y. J. Sun, J. T. Hu, P. J. Ni and Z. Li, *Analyst*, 2013, 138, 7152–7156.

8 Z. Li, B. Zhao, D. Wang, Y. Wen, G. Liu, H. Dong, S. Song and C. Fan, *ACS Appl. Mater. Interfaces*, 2014, *6*, 17944–17953.
